# A novel mini-invasive step-up approach for the treatment of severe acute pancreatitis with extensive infected necrosis: A single center case series study

**DOI:** 10.1097/MD.0000000000033288

**Published:** 2023-03-17

**Authors:** Beiyuan Zhang, Tao Gao, Yan Wang, Hao Zhu, Song Liu, Ming Chen, Wenkui Yu, Zhanghua Zhu

**Affiliations:** a Department of Critical Care Medicine, Nanjing Drum Tower Hospital, The Affiliated Hospital of Nanjing University Medical School, Nanjing, Jiangsu Province, China; b Department of Gastroenterology, Nanjing Drum Tower Hospital, The Affiliated Hospital of Nanjing University Medical School, Nanjing, Jiangsu Province, China; c Department of Radiology, Nanjing Drum Tower Hospital, The Affiliated Hospital of Nanjing University Medical School, Nanjing, Jiangsu Province, China.

**Keywords:** infection of pancreatic necrosis, lumen-apposing metal stent, percutaneous endoscopic necrosectomy, step-up approach, transgastric necrosectomy

## Abstract

Complete removal of necrosis is critical for treating patients with severe acute pancreatitis (SAP) presenting infection of pancreatic necrosis (IPN). Frequently used mini-invasive methods include the surgical step-up approach suitable for necrosis extending laterally, whereas the endoscopic step-up approach is suitable for medial necrosis. However, in patients with extensive IPN, either approach alone usually has limited treatment effects.

**Patient concerns:**

We describe a case series of combined mini-invasive step-up approach for treating extensive IPN.

**Diagnoses:**

Patients were diagnosed with SAP and had extensive IPN.

**Interventions:**

Seven patients with SAP and extensive IPN were enrolled. All patients underwent a combined step-up approach comprising 4 steps: percutaneous catheter drainage, continuous negative pressure irrigation (CNPI), percutaneous endoscopic necrosectomy (PEN), and transgastric necrosectomy (TN).

**Outcomes:**

The median interval from symptom onset to percutaneous catheter drainage and CNPI was 11 days (range, 6–14) and 18 days (range, 14–26), and the median CNPI duration was 84 days (range, 54–116). The median interval from the onset of symptoms to PEN and TN was 36 days (range, 23–42) and 41 days (range, 34–48), respectively, and the median number of procedures was 2 (range, 1–2) for PEN and 3 (range, 2–4) for TN. Only a minor case of abdominal bleeding and a pancreatic-cutaneous fistula were reported, both resolved after conservative treatment. The median length of stay in the intensive care unit was 111 days (range, 73–133); all patients survived.

**Lessons:**

This mini-invasive step-up approach shows promising clinical effects and is relatively safe in critically ill patients with extensive IPN and high-risk surgical intervention.

## 1. Introduction

Severe acute pancreatitis (SAP), defined as persistent organ failure for more than 48 hours according to the revised Atlanta classification, is the most severe form of acute pancreatitis (AP), with up to 20% to 40% of cases presenting necrotic infections.^[1,2]^ Infection with pancreatic necrosis (IPN) is a decisive factor defining the severity of the disease.^[3]^ The mortality of patients with IPN is substantially higher than that of patients without IPN, ranging from 14% to 69% in various series.^[4]^

A variety of minimally invasive techniques, including percutaneous catheter drainage (PCD), endoscopy, laparoscopy, and retroperitoneal approaches, have emerged and have gained popularity in the treatment of patients with IPN in recent decades. These techniques can further reduce surgical stress, and some do not require general anesthesia, thereby reducing complications.^[5–8]^ Current guidelines recommend step-up approaches combining these new techniques sequentially as an initial invasive treatment for necrotizing pancreatitis.^[2,9]^ There are 2 commonly used methods, surgical and endoscopic step-up approaches: the first involves the treatment of PCD followed by video-assisted retroperitoneal debridement (VARD) or endoscopic necrosectomy through the sinus tract, and the latter involves initial transgastric drainage and subsequent necrosectomy through the gastric wall using endoscopic ultrasound guidance if necessary.^[10]^ PCD has always been considered the principal treatment for patients with fluid collections or necrosis that extends to deeper anatomical planes in the AP at an early stage (<4 weeks).^[11]^ Affected by the trajectory of PCD, the surgical step-up approach is mainly applicable to lateral fluid collection or necrosis (such as paracolic gutters, pararenal space, and pelvis), while its effects are limited for fluid collections or necrosis confined to the vicinity of the gastroduodenal location, such as the posterior gastric wall and lesser sac, and the endoscopic step-up approach could be more suitable in such cases. Therefore, a combined approach, using both techniques performed by multidisciplinary teams, is advocated for the treatment of patients with extensive IPN to combine the benefits of both step-up approaches.

In this study, we developed a novel mini-invasive step-up approach consisting of 4 steps, including percutaneous endoscopic necrosectomy (PEN) combined with transgastric necrosectomy (TN). To further optimize the step-up scheme, a lumen-apposing metal stent (LAMS), which has a large diameter for facilitating the operation and thus reducing the operation time, was used in TN because it is more suitable in severely ill patients.^[12]^ In this case series, we demonstrated the feasibility and outcome of this combined minimally invasive approach for the treatment of extensive IPN in patients with SAP.

## 2. Methods

### 2.1. Patients

**Figure 1.**
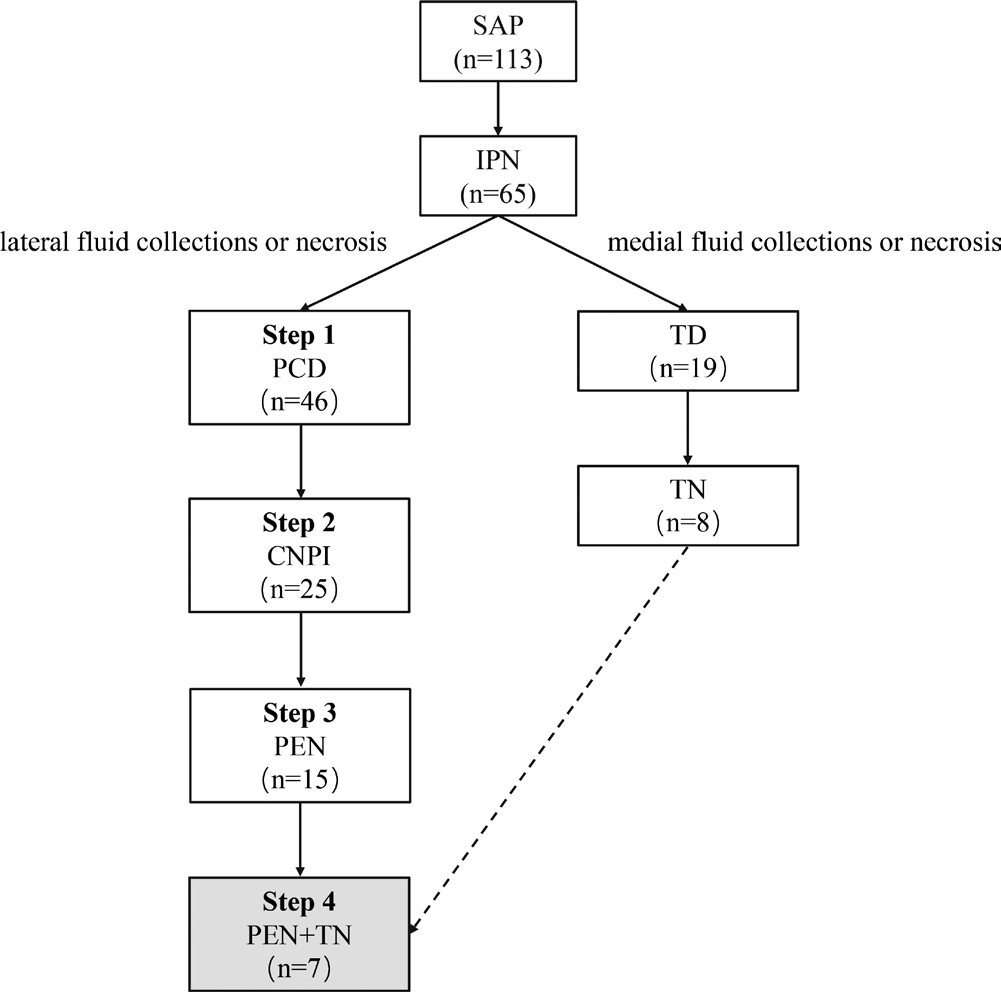
Flow chart. The dotted line means no patient. CNPI = continuous negative pressure irrigation, IPN = infection of pancreatic necrosis, PCD = percutaneous catheter drainage, PEN = percutaneous endoscopic necrosectomy, SAP = severe acute pancreatitis, TD = transgastric drainage, TN = transgastric necrosectomy.

A total of 113 patients with SAP were admitted to our center (a tertiary teaching hospital) between January 2019 and January 2022. Of these, 65 were diagnosed with IPN. Seven patients each received PEN and TN. We retrospectively analyzed a cohort of 7 patients with extensive IPN who were successfully treated using the mini-invasive 4-step approach. A flow chart is shown in Figure 1. AP was defined as acute abdominal pain in combination with serum amylase level ≥ 3 times the upper limit of normal with characteristic findings on abdominal computed tomography (CT).^[1]^ SAP was diagnosed according to the revised Atlanta Classification, which defines simultaneous persistent single or multiple organ failure > 48 hours based on AP diagnosis.^[1]^ Pancreatic necrosis is characterized by non-enhancement compared to normal pancreatic parenchyma on contrast-enhanced CT images. IPN was considered when the following conditions occurred: persistent fever ≥ 38.5ºC; increased white blood cells count, C-reactive protein, or procalcitonin levels; rapid deterioration of clinical condition; and signs of gas present in areas of necrosis on CT images.^[13]^ Although the diagnosis of IPN depends on fine-needle aspiration, and culture, it was not applied in our study because clinical and imaging signs are accurate predictors of IPN in most patients. The study protocol was approved by the Medical Ethics Committee of Nanjing Drum Tower Hospital (number:2021-147-01). This study was registered in the Clinical Trial Registry (www.clinicaltrials.gov) and the identifier was NCT05508828.

### 2.2. SAP initial treatment protocol

In the first few days after admission, patients received intravenous analgesics, omeprazole, and somatostatin, and enteral nutrition was administered through nasojejunal tubes whenever possible. Traditional Chinese medicine was administered via tube feeding or enema to improve gastrointestinal function. Prophylactic anti-infective therapy was not routinely administered, but broad-spectrum antibiotics were administered empirically once the patient developed fever. If the patients had concomitant organ failure, appropriate organ support was provided, including restrictive fluid resuscitation, use of vasoactive agents, continuous renal replacement therapy, and invasive mechanical ventilation. Patients with biliary pancreatitis underwent endoscopist–specialist consultations and endoscopic retrograde cholangiopancreatography within 72 hours after admission. For patients with hyperlipidemia and plasma triglyceride ≥ 11.3 mmol/L, and plasma exchange was administered immediately. Once the patient presented persistent fever, jaundice, and gallbladder enlargement, which was assessed using abdominal CT or ultrasound, ultrasound-guided percutaneous transhepatic gallbladder drainage was performed under local anesthesia. All patients underwent contrast-enhanced CT within 48 hours after admission to hospital identify the location and range of necrosis and to calculate the CT severity index (CTSI).

### 2.3. Novel mini-invasive step-up approach

In contrast to conventional step-up approaches, our mini-invasive step-up approach consisted of 4 procedures: PCD, continuous negative pressure irrigation (CNPI), PEN, and TN.

#### 1.2.3. Step one: PCD

If the patient’s condition progressively deteriorated after admission, CT or ultrasound-guided drainage was performed with a central venous catheter (Baihe, Baihe Medical Technology Co., Ltd., Guangdong, China) or pigtail catheter (BIOTEQ, Bioteque Corporation, Taiwan, China) using a 0.032 stiff guidewire, based on the location of necrosis. Pus samples were withdrawn and sent for culture and microbial sensitivity testing. The catheters were flushed with 5 to 10 mL of 0.9% normal saline to ensure smooth drainage. If the IPN evaluated by abdominal CT was not adequately drained or a gastrointestinal/pancreatic fistula was confirmed or suspected, a second step was applied.

#### 2.2.3. Step two: CNPI

**Figure 2.**
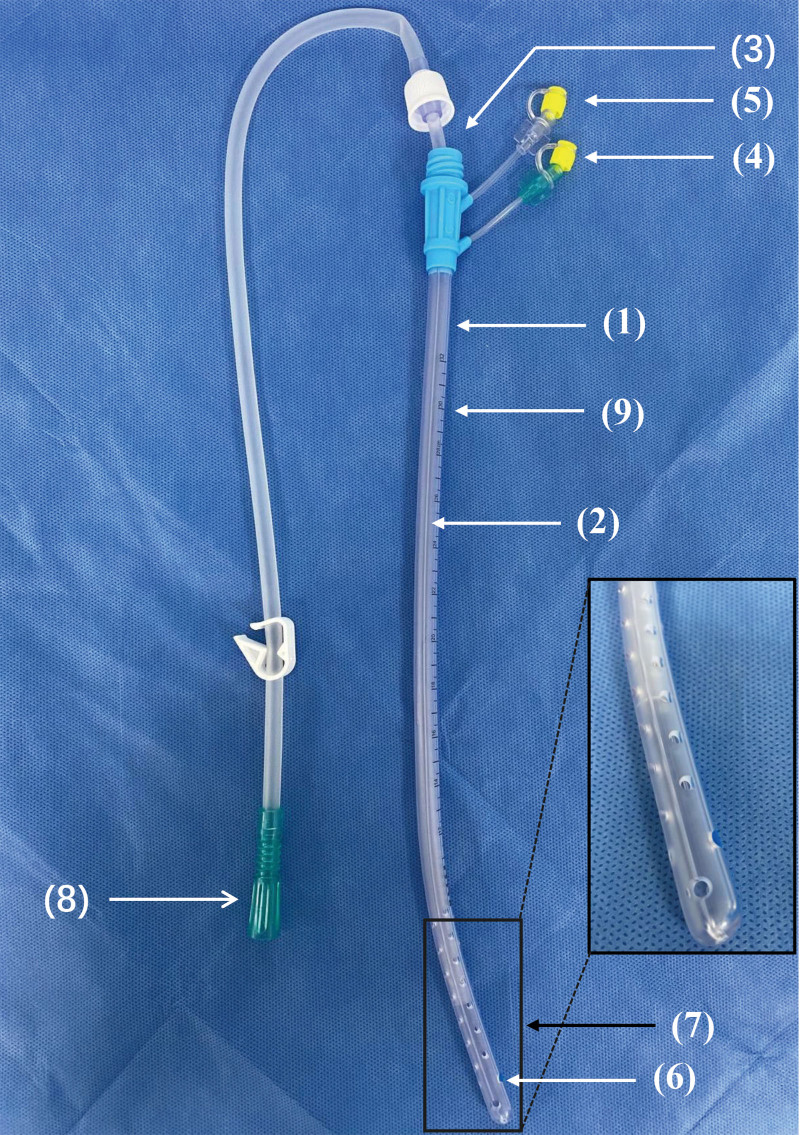
The structure of multifunction drainage tube: (1) the outer tube; (2) the capillary tube; (3) the inside drainage tube; (4) the drug entrance; (5) the water entrance; (6) drainage holes; (7) the headend; (8) the adapter; (9) scale mark. The capillary tube is nested in the wall of the outer tube up to the headend which contains many drainage holes, and the main function is continuous irrigation of the cavity through the drug entrance. The inside drainage tube is inside the outer tube and connected to a negative pressure device through the adapter. The water entrance is for adjust the pressure to ensure the patency of inside drainage tube.

A multifunctional drainage tube (Waylight, Sunlight Medical Co., Ltd., Guangdong, China) was used for CNPI. The tube structure consisted of 3 parts: an outer tube, an inner drainage tube, and a capillary tube. The head of the outer tube (the headend) was placed in the necrotic cavity and there were several drainage holes on the wall, each with a diameter of 3 to 5 mm. The capillary tube was nested in the wall of the outer tube up to the headend, and its main function was the continuous irrigation of the cavity. The drainage tube was inside the outer tube, and the extension part of its end (the adapter) was connected to a negative pressure device. Two small holes (approximately 3 mm in diameter), named the drug entrance and the water entrance, were set parallel to the outer tube wall. The drug entrance was used as the inlet of the capillary tube through which the liquid reached the cavity along the capillary tube for flushing. The entrance of the water was used mainly to adjust the pressure to ensure the patency of the inside drainage tube. The sizes of the multifunction drainage tubes used in our study were M6A, M8A, or M10A. The corresponding lengths of the outer tubes were 320, 350, and 360 mm, and the diameters were 6.0, 8.0 mm and 10.0 mm, respectively. A multifunction drainage tube was placed along the previous percutaneous sinus tract which expanded using 18 to 30 Fr dilators or was placed using the CT-guided Seldinger technique for the newly emerging necrotic cavity. The procedures were similar to that of PCD described above. After surgery, a large-volume of 0.9% saline solution was slowly infused into the necrotic cavity (1500–2000 mL/day) through the drug entrance, allowing for continuous irrigation (Fig. 2). All tubes were checked daily and replaced when they were translocated or heavily blocked.

#### 3.2.3. Step three: PEN

If necrosis was not effectively removed based on abdominal CT images or if the patient’s condition continued to deteriorate, repeated PEN was performed by 1 or 2 experienced endoscopists under conscious sedation. An electronic gastroscope (30F) was passed through the percutaneous sinus tract to remove solid necrosis with the help of a snare.

#### 4.2.3. Step four: TN

**Figure 3.**
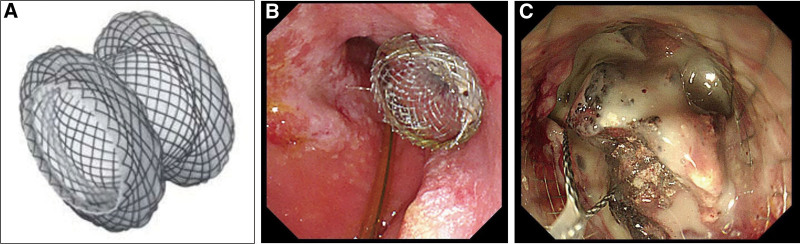
Endoscopic transgastric necrosectomy in patient 4. (A) LAMS, (B) endoscopic image of the inserted and unfolded LAMS, and (C) endoscopic necrosectomy using a snare through the LAMS. LAMS = lumen-apposing metal stent.

**Figure 4.**
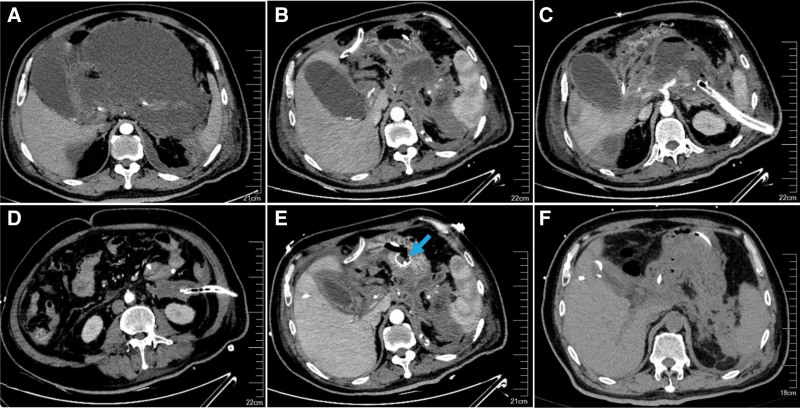
CT images of the abdomen in patient 4. (A) An initial CT scan shows extensive pancreatic necrosis and peripancreatic fluid collection, with a CTSI of 8, (B–D) CT-guided multifunction drainage tubes were placed through previously established percutaneous sinus tract in the perihepatic space, left anterior pararenal space and paracolic gutter at day 14 after PCD were performed. Subsequently, CNPI was initiated through the multifunction drainage tubes, (E) PEN and TN using LAMS was performed simultaneously at day 15 after CNPI were performed. The LAMS was seen in the wall of the stomach (blue arrow), and (F) CT scan was performed at 91 days after the onset of the disease and the images shows that the pancreatic and peripancreatic necrosis was fully removed. CT = computed tomography, CTSI = CT severity index, CNPI = continuous negative pressure irrigation, LAMS = lumen-apposing metal stent, PCD = percutaneous catheter drainage, PEN = percutaneous endoscopic necrosectomy, TN = transgastric necrosectomy.

TN was performed subsequently or simultaneously by the same endoscopist if the patient’s condition did not improve after PEN. Briefly, the location of necrosis and its distance from the gastric wall were evaluated using linear endoscopic ultrasound (EUS), and the optimal puncture path was determined. The LAMS (Hot AXIOS^TM^ Stent and Electrocautery Enhanced Delivery System; Boston Scientific Corporation, Galway, Ireland), which is a fully covered self-expandable metal stent with a diameter of 15 mm and length of 10 mm, was used in our study. The necrotic cavity was punctured using an electrocautery tip, and the delivery catheter was advanced into the cavity. The distal flange of the metal sent was deployed under EUS guidance, then the stent was retracted and aligned, and finally the proximal flange was released. The bulk of necrosis and debris were dragged out with a snare through the sent, and the necrotic cavity was flushed with 0.9% saline solution. TN was repeated, mostly every 3 to 5 days, until all loosely adherent necrosis was cleared and replaced by granulation tissue (Fig. 3). The stent was removed approximately 4 to 6 weeks after implantation. Subsequently, the CNPI was stopped and replaced with simple drainage if the patient’s condition continues to improve and the cavity was resolution evaluated by abdominal CT. Once the drainage volume was <10 mL/day for 3 consecutive days, the drainage tube was clamped and finally removed (Fig. 4).

### 2.4. Data collection

The following data were collected: age, sex, etiology of pancreatitis, CTSI score at admission, new-onset organ failure during hospitalization, acute physiology and chronic health evaluation II, sequential organ failure assessment (SOFA) score within the first 24 hours of admission to the intensive care unit (ICU), results of the microbiological culture of drainage fluid and necrosis, and details of each step, including time, frequency and duration of each procedure. Complications associated with the procedure including intra-abdominal bleeding, gastrointestinal bleeding, gastrointestinal/pancreatic fistula, length of ICU stay, and prognosis at hospital discharge were also recorded.

## 3. Results

Of the 7 patients, enrolled in the study, 2 were male and 5 were female, and the median age was 48 years (range, 32–71). Three patients were diagnosed with biliary pancreatitis, 3 with hyperlipidemic pancreatitis, and 1 with idiopathic pancreatitis. One patient underwent endoscopic retrograde cholangiopancreatography and 2 patients underwent plasma exchange within 72 hours after admission to the ICU. The median acute physiology and chronic health evaluation II, SOFA, and CTSI scores were 20 (range, 16–27), 11 (range, 7–14), and 8 (range, 8–10), respectively. New-onset organ failure during hospitalization was respiratory in nature in 5 patients, cardiovascular in 3, and renal in 4. Furthermore, 3 patients received invasive mechanical ventilation, 3 received fluid resuscitation and vasoactive drugs, and 4 received continuous renal replacement therapy (Table 1). The median time interval from the onset of the symptoms to the first PCD procedure was 11 days (range, 6–14 days). The median time interval from the onset of the symptom to the CNPI was 18 days (range, 14–26 days). The median number of multifunction drainage tubes placed during the ICU stay per patient was 3 (range, 2–4), and the median CNPI duration was 84 days (range, 54–116). The median time interval from symptom onset to PEN and TN was 36 days (range, 23–42) and 41 days (range, 34–48), respectively, and the median number of procedures was 2 (range, 1–2) for PEN and 3 (range, 2–4) for TN (Table 2). In addition, patient 4 received PEN and TN simultaneously on day 41 after the onset of the disease due to persistent malignant hyperthermia and organ dysfunction.

**Table 1:** Patient characteristics.

Patient/gender	Age, yr	Etiology	APACHE II score	SOFA score	CTSI score	New-onset organ failure	Complications	ICU stay, days	Outcome
1/F	54	Biliary	19	11	8	Respiratory	Abdominal bleeding	73	Survival
2/M	46	Hyperlipemia	20	12	10	Cardiovascular, renal	None	129	Survival
3/F	35	Hyperlipemia	16	7	8	Respiratory, renal	None	127	Survival
4/M	48	Idiopathic	18	8	8	Renal	None	83	Survival
5/F	71	Biliary	27	14	10	Respiratory, cardiovascular	None	79	Survival
6/F	58	Biliary	25	13	8	Respiratory, cardiovascular	Pancreatic-cutaneous fistula	133	Survival
7/F	32	Hyperlipemia	23	11	10	Respiratory, renal	None	111	Survival

APACHE II = acute physiology and chronic health evaluation II, CTSI = computed tomography severity index, ICU = intensive care unit, SOFA = sequential organ failure assessment.

**Table 2:** Details of the therapy regime and pathogen of drainage liquid or necrosis cultures.

Patient	Time to PCD*, days	Time to CNPI†, days	Number of tubes‡	Days of CNPI, days	Time to PEN§, days	Number of PEN	Time to TNǁ, days	Number of TN	Cultured pathogen
1	6	14	4	57	39	2	43	4	*Enterococcus faecalis*
2	12	18	3	116	42	2	48	2	*Klebsiella pneumoniae Escherichia coli Proteus mirabilis*
3	9	15	4	110	31	2	38	3	*Enterococcus faecalis Klebsiella pneumoniae* *Escherichia coli*
4	12	26	4	65	41	1	41	2	*Klebsiella pneumoniae*
5	11	16	3	54	36	2	45	3	*Enterococcus faecalis Pseudomonas aeruginosa Acinetobacter baumannii*
6	14	23	2	109	30	1	37	2	*Enterococcus faecalis Klebsiella pneumoniae*
7	10	18	3	84	23	1	34	3	*Escherichia coli Enterococcus faecium*

CNPI = continuous negative pressure irrigation, PCD = percutaneous catheter drainage, PEN = percutaneous endoscopic necrosectomy, TN = transgastric necrosectomy.

* Time interval from symptoms onset to first PCD.

† Time interval from symptoms onset to CNPI.

‡ Number of multifunction drainage tube placed.

§ Time interval from symptoms onset to PEN.

ǁ Time interval from symptoms onset to TN.

The most common pathogens isolated from drainage fluid or necrosis cultures were *Enterococcus faecalis, Klebsiella pneumoniae*, and *Escherichia coli.* Two patients developed bacteremia during subsequent treatment, and the pathogen was consistent with the culture results of drainage liquid or necrosis indicating *K pneumoniae*, and *E faecalis* (Table 2).

No serious complications related to the procedure occurred during the course of treatment. In Patient 1, minor abdominal bleeding was observed on the second day after the placement of the multifunction drainage tube, which stopped soon after receiving conservative treatment, which included blood transfusions and discontinuation of irrigation. In patient 6, a pancreatic-cutaneous fistula developed because the peripancreatic drainage fluid turned greenish on the third day after TN, and the amylase level of the drainage fluid was 27,434 U/L. After 93 days of CNPI, the fistula healed spontaneously, without additional intervention. The median length of stay in the ICU was 111 days (range, 73–133) and none of the patients died (Table 1).

## 4. Discussion

In this study, we combined PEN and TN and proposed a novel step-up approach to the treatment of extensive IPN in patients with SAP. The patients enrolled in our study were severely ill, as evidenced by the high acute physiology and chronic health evaluation II and SOFA scores, and had extensive pancreatic/peripancreatic necrosis, as indicated by the CTSI score. Despite this, they were successfully managed with this integrated mini-invasive strategy without serious complications and avoided further open necrosectomy.

The surgical step-up approach, which initially involves PCD followed by VARD or endoscopic necrosectomy through the percutaneous sinus tract, was found to be superior to open necrosectomy.^[5,14]^ The PANTER study prospectively enrolled 88 patients with necrotizing pancreatitis, 24 of whom underwent percutaneous drainage and VARD; The results showed that the incidence of new-onset multiple organ failure, incisional hernias, and new-onset diabetes was significantly lower in patients treated with the surgical step-up approach than in those treated with open necrosectomy, although mortality did not differ between the 2 groups.^[5]^ Liu et al^[14]^ conducted a retrospective cohort study involving 27 patients with IPN suffering from failure PCD, 15 of whom subsequently underwent double-catheter lavage and PEN, and 12 underwent open necrosectomy. The authors concluded that the rate of new-onset multiple organ failure and the length of ICU stay were significantly lower in the PEN group than in the open necrosectomy group. The surgical step-up approach is ideal for treating patients with lateral fluid collections or necrosis that extends into the paracolic gutters, the pararenal space, and the pelvis, while the endoscopic step-up approach is suitable for patients with medial fluid collections or necrosis, that is, fluid collections or necrosis confined to the vicinity of the gastroduodenal location, such as the posterior gastric space and the lower sac. The endoscopic transgastric approach presents a lower risk of pancreatic fistula and a shorter hospital and ICU stay than surgical necrosectomy.^[15]^ However, only a few clinical studies have compared these 2 mini-invasive step-up approaches, and even fewer have reported on their combination. The PENGUIN trial prospectively enrolled 20 patients with IPN; endoscopic transgastric necrosectomy was performed in 10 patients, and VARD or open necrosectomy was performed in another 10 patients.^[7]^ The results showed that the former technique can further reduce the level of serum interleukin 6 and major complications, including new-onset multiple organ failure and pancreatic fistula. However, this result should be interpreted with caution due to the small sample size. Van Brunschot et al^[10]^ conducted a randomized clinical trial investigating the endoscopic *vs* surgical step-up approach in a cohort of 98 patients with necrotizing pancreatitis, who were randomly assigned to the 2 treatment arms. The authors found that the main complications, including bleeding, perforation of the visceral organs, enterocutaneous fistula, and incisional hernia, and mortality rates did not differ between the 2 groups, although the incidence of pancreatic fistula and length of hospital stay were lower in the endoscopic step-up approach group. The same group published another large sample study involving 1980 patients with original study data and unpublished data to compare mini-invasive surgical, endoscopic, and open necrosectomy for the treatment of necrotizing pancreatitis.^[16]^ The authors used propensity score matching with risk stratification to control possible confounders and found that both mini-invasive surgical and endoscopic necrosectomy were associated with a lower risk of death than open necrosectomy in the very high-risk group (predicted risk of death ≥ 35%). Furthermore, the effect of mini-invasive surgical necrosectomy on mortality was not different from that of endoscopic necrosectomy.

In 2016, Fagenholz et al^[17]^ first reported a case of extensive pancreatic necrosis complicated by enteric fistulae successfully treated using transgastric drainage combined with the VARD method. Sorrentino et al^[18]^ subsequently reported a patient with central walled-off pancreatic necrosis extending laterally to the bilateral retroperitoneal spaces complicated by multiorgan failure and choledochal fistula. The patient was successfully treated using a combined mini-invasive approach, including endoscopic transgastric necrosectomy, PCD, VARD, and endoscopic biliary stenting. In 2022, Lindgaard et al^[19]^ described 2 patients with large and complex walled-off pancreatic necrosis who underwent endoscopic transluminal drainage, endoscopic necrosectomy, and VARD using a laparoscopic access platform. After 34 days and 86 days of treatment and a total of 9 and 14 procedures, respectively, the walled-off pancreatic necrosis completely regressed in both cases.

There are 5 aspects of this approach that require further exploration. First, both PEN and TN have their individual application indications; this combined approach is suitable for patients with extensive necrosis, especially those with medial and lateral necrosis. Second, there is no relevant research on the timing and sequence of implementation of these 2 necrosectomy technologies. Despite this, we advocate the surgical step-up approach as a priority for patients with extensive IPN. The literature reports that approximately 13.6% to 17.0% of patients with SAP die in the early stages (first 2 weeks) due to excessive systemic inflammatory response syndrome and organ dysfunction.^[20,21]^ Early administration of PCD to relieve the systemic inflammatory response and improve organ function, followed by PEN through the sinus tract, has become the most common approach in clinical practice.^[22,23]^ Furthermore, critically ill patients, especially unstable patients, are more tolerant to this approach than to the transgastric approach. More importantly, this also provides sufficient time for the formation of walled-off necrosis. Until then, TN could carry a high risk of pneumoperitoneum or pneumoretroperitoneum, which may result in infective complications.^[24]^ Third, the CNPI was a bridge between PCD and PEN in our mini-invasive step-up approach. In recent years, large-bore percutaneous drainage tubes placed using the Seldinger technique and large-volume irrigation have been reported for the treatment of necrotizing pancreatitis.^[23,25]^ On the 1 hand, continuous lavage with 0.9% saline solution may provide a gentle way to clear the infection, thus improving necrosis cavity healing.^[26,27]^ Conversely, negative pressure treatment has been proven to be effective in removing necrotic debris and preventing re-accumulation of purulence in the wound bed, resulting in increased epithelial regeneration and granulation tissue coverage in the wound.^[28,29]^ Furthermore, Gao et al^[30]^ performed a retrospective analysis of 132 patients with IPN and colonic fistula who received a step-up approach including CNPI and found that the approach could avoid the need for subsequent surgery in 47% of patients, spontaneous closure of the fistula in 92% of patients, and in-hospital mortality fell to only 19%. The scavenging effect of CNPI on necrosis caused the frequency of PEN (range, 1–2) to be lower than that of TN (range, 2–4) in our study, resulting in a lower risk of external fistula formation. Fourth, TN was performed with LAMS in all our patients due to its large diameter (15 mm) and bi-flanged design, which facilitates endoscopic transluminal necrosectomy, reduces the risk of stent occlusion, and prevents stent migration. A large retrospective study enrolled 189 patients with pancreatic walled-off necrosis who underwent EUS-guided transmural drainage, of whom 102 had LAMS and 87 had plastic stent, respectively.^[31]^ Patients with LAMS had a higher clinical success rate, shorter procedure time, and a lower probability of requiring surgery and recurrence than those with plastic stents. However, 2 recent prospective studies evaluating the clinical effects and associated complications of LAMS have shown mixed results.^[32,33]^ In a randomized controlled trial by Bang et al^[32]^ involving 60 patients with pancreatitis and walled-off necrosis, patients with LAMS had similar technical success, treatment success, and the total number of procedures performed during the 6 months of follow-up was comparable to that obtained following placement of plastic stents. However, the endoscopic operation time was shorter in patients with LAMS despite the higher incidence of complications and costs. In contrast, another prospective cohort study enrolled 53 patients with IPN and found that the number of endoscopic transluminal necrosectomy procedures, incidence of complications, and total healthcare costs were comparable between the 2 stents.^[33]^ The results of 2 ongoing randomized controlled studies will further evaluate the clinical efficacy and possible complications^[34,35]^; however, shorter endoscope operation times with LAMS are unambiguously evident, which is a potentially crucial advantage for more critical cases.^[12,32]^ Finally, approximately 27% to 32% of patients with IPN who underwent the endoscopic step-up approach required additional PCD procedures.^[10,33]^ This may be due to the destruction of the well-defined necrosis wall associated with the endoscopic transluminal procedure, which results in the spread of necrosis, digestive enzymes, and pro-inflammatory mediators to other parts of the abdominal cavity. Furthermore, the endoscopic transluminal procedure also causes loss of retroperitoneal compartmentalization, leading to spread of necrosis into communicating recesses and cavities. Some studies have reported the use of a dual modality, which refers to the administration of PCD followed by an immediate endoscopic transluminal procedure in the treatment of necrotizing pancreatitis, and showed improved clinical results and a decreased need for surgical intervention.^[36,37]^ For our patients, the CNPI was maintained until all endoscopic procedures were complete. Only patient 6 experienced additional small-caliber PCD on day 3 after the endoscopic procedures because pelvic effusion was found on reexamined abdominal CT images.

## 5. Conclusions

Taken together, the proposed mini-invasive step-up approach combined PEN with TN in patients with SAP and extensive IPN. We believe that this novel strategy can effectively remove solid infected necrosis without serious complications, and no patient underwent surgical intervention. The disadvantage of this approach is that multiple catheterizations, as well as repeated PEN and TN procedures, are still required, resulting in prolonged hospitalization and relatively high costs. Further randomized controlled trials with larger sample sizes are needed to verify the clinical efficacy and safety of this approach.

## Acknowledgements

This research was supported by the National Natural Science Foundation of China (no. 81927808).

## Author contributions

**Conceptualization:** Beiyuan Zhang, Zhanghua Zhu, Wenkui Yu.

**Data curation:** Beiyuan Zhang, Tao Gao, Yan Wang.

**Funding acquisition:** Wenkui Yu.

**Investigation:** Ming Chen.

**Methodology:** Beiyuan Zhang.

**Resources:** Hao Zhu, Song Liu.

**Supervision:** Wenkui Yu.

**Writing – original draft:** Beiyuan Zhang.

**Writing – review & editing:** Zhanghua Zhu, Wenkui Yu.
